# The Dose–Response Decrease in Heart Rate Variability: Any Association with the Metabolites of Polycyclic Aromatic Hydrocarbons in Coke Oven Workers?

**DOI:** 10.1371/journal.pone.0044562

**Published:** 2012-09-14

**Authors:** Xiaohai Li, Yingying Feng, Huaxin Deng, Wangzhen Zhang, Dan Kuang, Qifei Deng, Xiayun Dai, Dafeng Lin, Suli Huang, Lili Xin, Yunfeng He, Kun Huang, Meian He, Huan Guo, Xiaomin Zhang, Tangchun Wu

**Affiliations:** 1 Department of Occupational and Environmental Health and Ministry of Education Key Lab for Environment and Health, School of Public Health, Tongji Medical College, Huazhong University of Science and Technology, Wuhan, China; 2 Institute of Industrial Health, Wuhan Iron and Steel (Group) Corporation, Wuhan, China; College of Tropical Agriculture and Human Resources, University of Hawaii, United States of America

## Abstract

**Background:**

Air pollution has been associated with an increased risk of cardiopulmonary mortality and decreased heart rate variability (HRV). However, it is unclear whether coke oven emissions (COEs) and polycyclic aromatic hydrocarbons (PAHs) are associated with HRV.

**Objectives:**

Our goal in the present study was to investigate the association of exposure to COEs and the urinary metabolite profiles of PAHs with HRV of coke oven workers.

**Methods:**

We measured benzene soluble matter, carbon monoxide, sulfur dioxide, particulate matters, and PAHs at different workplaces of a coke oven plant. We determined 10 urinary PAH metabolites and HRV indices of 1333 workers using gas chromatography–mass spectrometry and a 3-channel digital Holter monitor, respectively.

**Results:**

Our results showed that there was a significant COEs-related dose-dependent decrease in HRV, and an inverse relationship between the quartiles of urinary 2-hydroxynaphthalene and five HRV indices (*p*_trend_<0.01 for all). After adjustment for potential confounders, elevation per interquartile range (IQR) (1.81 µg/mmol creatinine) of urinary 2-hydroxynaphthalene was associated with a 5.46% (95% CI, 2.50–8.32) decrease in standard deviation of NN intervals (SDNN). As workers worked more years, SDNN gradually declined in the same quartiles of 2-hydroxynaphthalene levels (*p*_trend_ = 1.40×10^−4^), especially in workers with the highest levels of 2-hydroxynaphthalene.

**Conclusions:**

Occupational exposure to COEs is associated with a dose-response decrease in HRV. In particular, increased exposure to 2-hydroxynaphthalene is associated with significantly decreased HRV. Increase of working years and exposure levels has resulted in a gradual decline of HRV.

## Introduction

Evidence is accumulating that air pollution is associated with an increased risk of cardiovascular mortality and morbidity [1]–[4], but the potential biologic mechanisms underlying such effects remain to be elucidated. Heart rate variability (HRV) reflects autonomic modulation of the rhythmic activity of the sinus node and is analyzed in the time or frequency domains [5]. Altered HRV can be used to assess cardiac autonomic function, which is one of the pathophysiologic pathways involved in various cardiovascular events [1]. Consistent associations between decreased HRV and adverse health outcomes such as hypertension [6], ischemic heart disease [7], coronary artery disease [8], [9], congestive heart failure [9], [10], sudden cardiac death [11] and myocardial infarction [9], [12] have already been observed in previous studies. In particular, reduced HRV has been identified as an independent predictor for evaluating the cardiovascular effects associated with ambient air pollution [5], [9], [12]–[15]. Ambient air pollution is a complex mixture of gases, liquids, and particulate matter (PM) and consists of various constituents, mainly from traffic-related and coal-combustion-related pollutants. Several studies have shown that traffic-related pollutants result in reduced HRV [16]–[19]. Cavallari et al. (2007) reported that occupational exposure to oil-combustion-related and construction-related pollutants was associated with decreased HRV [20]. The combustion of coal is a major contributor of these pollutants in urban areas, especially in developing countries; however, there are few studies about the specific effects of coal-combustion-related pollutants on HRV.

A coke oven plant includes facilities for coal storage, coke sieving, coking and coke oven gas purification. Coke oven workers are at high risk of exposure to coke oven emissions (COEs) during the production of coke. COEs are complex coal-combustion-related air pollutants which contain various toxic chemical substances, such as polycyclic aromatic hydrocarbons (PAHs), carbon monoxide (CO), and sulfur dioxide (SO_2_). PAHs are the main chemical components of COEs, which are commonly seen in occupational settings such as the pyrolysis of coal, fossil fuels, coke production, and in iron and steel foundries. COEs are also present in everyday life from traffic-related and smoke emissions. Occupational PAH exposure was associated with an exposure-response increase in both cancers and fatal ischemic heart disease [21]–[23]. Lee et al (2010) determined urinary 1-hydroxypyrene in 40 boilermakers exposed to oil-combustion-related pollutants and that decreased night time HRV was associated with urinary 1-hydroxypyrene [24]. However, no study has examined the association of urinary metabolite profiles of PAHs with HRV in coke-oven workers.

In the present study, we tested the hypothesis that occupational exposure to COEs and all urinary metabolites of PAHs are associated with decreased HRV. Therefore, we measured COEs (benzene soluble matter (BSM), PAHs, CO, SO_2_ and PM) at different workplaces, and all detectable urinary PAH metabolites and HRV indices (standard deviation of NN intervals (SDNN), root mean square of successive differences in adjacent NN intervals (rMSSD), low frequency (LF), high frequency (HF) and total power (TP)) of 1333 workers in a coke oven plant. The potential role of HRV as an indicator of increased cardiovascular disease risk will be very useful for providing guidelines for the early detection of damage to the cardiovascular system and for the improvement of health outcomes. Additionally, this study will further promote the prevention and control of occupational exposure to PAHs in workers, and will initiate further research on the possible mechanism of HRV decline caused by PAHs.

## Materials and Methods

### Ethics statement

The research protocol was approved by the Ethics and Human Subject Committee of Tongji Medical College, and written informed consent was obtained from all participants.

### Airborne COEs monitoring

The airborne samples were collected from different workplaces of coke oven plant as described previously [25]. The concentrations of PM in the working environment were analyzed gravimetrically. The filters were weighed by a microbalance at least three times before and after sampling in a humidity- and temperature- controlled room. A total of 16 PAHs from the collected PMs were determined by high- performance liquid chromatography with fluorescence detectors according to Method 5506 of the U.S. National Institute for Occupational Safety and Health [26]. The other pollutants of COEs were detected using the benzene ultrasound elution method for BSM (GB, 17054-1997, China), an air quality- testing instrument for CO and the formaldehyde absorbing- pararosaniline spectrophotometry method for SO_2_ (HJ, 482-2009, China).

### Subjects and methods

We recruited 1333 coke oven workers from a coke oven plant in Wuhan (Hubei, China). They had been employed for at least one year and worked at the top, side, bottom, adjunct of coke ovens or in offices. Information on age, sex, smoking status, drinking habits, exercise, occupational location, length of employment, living conditions, and personal medical history was collected using standardized occupational questionnaires by trained reviewers. We excluded with serious medical conditions affecting HRV, such as heart failure, angina, arrhythmia, myocardial infarction and other nonclassified heart problems. A fasting blood sample was drawn for examination of cholesterol, triglycerides, high-density lipoprotein and low-density lipoprotein and blood glucose. Body height and weight were measured to compute the body mass index (BMI). With the subjects in a seated position, blood pressure was measured twice on the left upper arm. Hypertension was defined as either having a systolic blood pressure ≥140 mmHg or a diastolic blood pressure ≥90 mmHg or having been diagnosed with hypertension by a physician. In addition, morning urine samples (20 mL each) were collected in sterile conical tubes and stored at −20°C until laboratory analysis. The study design is presented in Figure S1.

### Determination of urinary PAH metabolites

We determined urinary PAH metabolites (1-hydroxynaphthalene, 2-hydroxynaphthalene, 2-hydroxyfluorene, 9-hydroxyfluorene, 1-hydroxyphenanthrene, 2-hydroxyphenanthrene, 3-hydroxyphenanthrene, 4-hydroxyphenanthrene, 9-hydroxyphenanthrene, 1-hydroxypyrene, 6-hydroxychrysene and 3-hydroxybenzo[a]pyrene) by gas chromatography-mass spectrometry described previously with some modifications [27]. Because 6-hydroxychrysene and 3-hydroxybenzo [a] pyrene were below the limits of quantification, we determined 10 metabolites of PAHs. Briefly, each 3.0 mL of urine was extracted three times to elevate the detection rate. A set of the standard curve was re-run for 100 urine samples. The identification and quantification of urinary PAH metabolites were based on retention time, mass-to-charge ratio and peak area using a linear regression curve obtained from separate internal standard solutions. The limits of detection (LOD) for the urinary PAH metabolites were in the range 0.1–1.4 µg/L; default values were replaced with 50% of the LOD. Valid urinary PAH metabolite concentrations were calibrated by levels of urinary creatinine and expressed as micrograms per mole of creatinine.

### Measurements of HRV

The HRV indices were measured after urine sample collection. After at least a 5-min rest, each participant was seated on a chair comfortably and was fitted with a 3-channel digital Holter monitor (Lifecard CF; Del Mar Reynolds Medical, Inc., Whitney, Irvine, USA) with a 1024 samples/second sampling rate for 10 minutes. We cleaned the participant's skin with an alcohol wipe and abraded it slightly to keep good lead contacts, and placed separate electrodes in position according to the instructions and technical manual of Lifecard CF (Del Mar Reynolds Medical, Inc., Whitney, Irvine, USA). The scanner collected automatically and all of the HRV indices were calculated on 5-min epoch in the entire recording. Only heart rates between 40 and 100 beats per minute were submitted to analyses [28]. We selected 5 consecutive minutes of ECG reading in the statistical analysis without atria and ventricular premature beats and flutter. The HRV spectrum was computed with a fast Fourier transform method. The HRV was analyzed in both time and frequency domains. The measured time domain parameters included: a) SDNN (standard deviation of all normal to normal NN intervals; in milliseconds), is an estimation of total HRV power; b) rMSSD (the root mean of square of successive differences between adjacent normal NN intervals; in milliseconds), reflects the activities of parasympathetic nervous system. The frequency-domain variables include: a) low-frequency (LF msec^2^; 0.04–0.15 Hz) power, may represent the combination of both parasympathetic and sympathetic activity of heart rate; b) high-frequency (HF msec^2^; 0.15–0.4 Hz) power, shows the actions of the parasympathetic modulation of heart rate; c) total power (TP msec^2^; approximately≤0.4 Hz) which is a mixture of the total variability of the heart rate [5].

### Statistical analyses

We assessed the normality of all variables with the one-sample K-S test. Normal distributions of HRV measures and the values of all urinary PAH metabolites were obtained by natural logarithmic transformation. We tested the age, working years, and BMI with one-way analysis of variance (ANOVA) for differences among different environmental exposure levels. We evaluated the categorical variables (such as sex, current smokers, alcohol use, exercise and hypertension) in different groups using the Chi-square test. The urinary PAH metabolite concentrations were categorized into quartiles. We performed multivariate analysis of covariance for the differences in the HRV parameters among quartiles of PAH metabolites with adjustment for age, sex, working year, smoking status, alcohol use, BMI, exercise and hypertension. Multivariate linear regression was performed for the trend of HRV with adjustment for the same variables. For the analyses stratified by internal exposure, we divided the participants into four groups using the quartiles of working years to estimate the joint effect of working years and exposure dose. All statistical tests were derived from two-sided analyses and *P*<0.05 was considered as statistical significant. The analysis was conducted using SPSS (version 12.0).

## Results

### Levels of COEs at different workplaces

Table 1 and Table S1 show the results of airborne monitoring of COEs including BSM, CO, SO_2_, and particulate matter with aerodynamic diameters ≤10 (PM_10_) and ≤2.5 (PM_2.5_) and PAHs at different workplaces in the coke oven plants. The concentrations (mean ± SD) of BSM, CO, SO_2_, PM_10_, PM_2.5_ and total PAHs were highest at the top of the coke oven (n = 15, 0.66±0.40, 23.00±7.61, 0.85±0.88, 2.65±1.74, 1.92±1.59 and 90.3±69.51, respectively), lower at the bottom and side (n = 16, 0.38±0.24, 3.19±1.51, 0.27±0.21, 0.84±0.49, 0.82±0.54 and 11.08±7.29, respectively), and lowest in the office (n = 5, 0.33±0.05, 1.50±0.81, 0.12±0.09, 0.26±0.19, 0.19±0.17 and 1.13±0.37, respectively). Based on the concentrations of airborne COEs, we defined office workers as the control group, and workers at adjunct workplaces, and those at the bottom, side, and top of the coke oven as low, intermediate and high exposure groups, respectively.

**Table 1:** Levels of environmental pollutants in different groups (mean ± SD).

Pollutants	Office(Control group, n = 5)	Coke oven (Exposure groups)		
		Adjunct workplaces(Low, n = 6)	Bottom and side(Intermediate, n = 16)	Top(High, n = 15)
BSM (mg/m^3^)	0.33±0.05	0.34±0.26	0.38±0.24	0.66±0.40
CO (mg/m^3^)	1.50±0.81	1.80±0.96	3.19±1.51	23.00±7.61
SO_2_ (mg/m^3^)	0.12±0.09	0.27±0.14	0.27±0.21	0.85±0.88
PM_10_ (mg/m^3^)	0.26±0.19	0.42±0.17	0.84±0.49	2.65±1.74
PM_2.5_ (mg/m^3^)	0.19±0.17	0.35±0.09	0.82±0.54	1.92±1.59
Total PAHs (µg/m^3^)	1.13±0.37	3.72±2.09	11.08±7.29	90.30±69.51

BSM: benzene soluble matter; CO: carbon monoxide; SO_2_: sulfur dioxide; PM: particulate matter; PAHs: polycyclic aromatic hydrocarbons.

### Characteristics and urinary PAH metabolites of workers

The general characteristics and urinary PAH metabolites of the workers in the control and in the low, intermediate, and high exposure groups are presented in Table 2 and Table 3. The distribution according to sex, current smoking habits, alcohol use and exercise in the four groups was different (*P*<0.05), and age, working years, BMI and hypertension were similar (*P*>0.05). Compared with the control group, nine urinary PAH metabolites (including 1-hydroxynaphthalene, 2-hydroxynaphthalene, 2-hydroxyfluorene, 9-hydroxyfluorene, 1-hydroxyphenanthrene, 2-hydroxyphenanthrene, 3-hydroxyphenanthrene, 9-hydroxyphenanthrene and 1-hydroxypyrene) were significantly higher in the three exposure groups, except for 4-hydroxyphenanthrene. There was a significantly increased trend of nine urinary PAH metabolites with increased environmental COEs levels (*P*_trend_<0.001, *P*_trend_<0.001, *P*_trend_<0.001, *P*_trend_ = 0.049, *P*_trend_<0.001, *P*_trend_<0.001, *P*_trend_<0.001, *P*_trend_<0.001, *P*_trend_ = 0.002 and *P*_trend_<0.001, respectively).

**Table 2:** General characteristics of the workers in different groups.

Characteristics	Control group(n = 470)	Exposure groups			*p*-Value
		Low(n = 496)	Intermediate(n = 265)	High(n = 56)	
Age (years, mean ± SD)	42.90±8.16	41.95±8.35	42.4±7.90	42.23±8.04	0.349*
Sex, male/female (% Male)	360/110 (76.6)	428/68 (86.3)	239/26 (90.2)	56/0 (100)	<0.001†
Working years (years, mean ± SD)	21.79±9.41	20.54±10.24	21.26±9.26	21.34±8.98	0.249*
Current smokers, yes/no (% yes)	239/231 (50.9)	279/217 (56.3)	165/100 (62.3)	42/14 (75.0)	0.001†
Alcohol users, yes/no (% yes)	148/322 (31.5)	159/337 (31.5)	103/162 (38.9)	28/28 (50.0)	0.010†
BMI (kg/m^2^, mean ± SD)	23.64±2.98	23.91±3.66	23.16±5.06	23.79±2.60	0.068*
Exercise, yes/no (% yes)	250/220 (53.2)	231/265 (46.6)	109/156 (41.1)	21/35 (37.5)	0.005†
Hypertension, yes/no (% yes)	53/417 (11.3)	49/447 (9.9)	37/228 (14.0)	6/50 (10.7)	0.408†
Cholesterol (mmol/l, mean ± SD)	4.70±0.92	4.7 9±0.89	4.91±0.90	4.78±0.88	0.054*
Triglycerides (mmol/l, mean ± SD)	1.78±1.99	1.76±1.66	1.88±2.22	1.64±0.98	0.807*
High-density lipoprotein (mmol/l, mean ± SD)	1.49±0.34	1.47±0.32	1.49±0.31	1.55±0.37	0.379*
Low-density lipoprotein (mmol/l, mean ± SD)	2.76±0.77	2.87±0.76	2.90±0.78	2.79±0.68	0.112*
Fasting blood glucose (mmol/l, mean ± SD)	5.96±1.23	5.81±0.86	6.00±1.75	6.38±2.14	0.015*

BMI: body mass index.

* One-way ANOVA for the differences between different exposure groups.

† Chi-square tests for the differences in the distribution frequencies between different exposure groups.

**Table 3:** Urinary PAH metabolites and HRV indices of the workers in different groups.

PAH metabolites or HRV	Control group	Exposure groups			*p*_trend_
		Low	Intermediate	High	
PAH metabolites (µg/mmol creatinine, mean ± SD)*					
1-hydroxynaphthalene	1.77±1.73	2.35±2.77	2.78±2.43	4.14±4.33	<0.001
2-hydroxynaphthalene	1.63±1.45	1.97±2.09	2.68±2.31	4.57±6.92	<0.001
2-hydroxyfluorene	0.89±0.67	1.36±1.90	1.39±1.31	2.17±2.20	<0.001
9-hydroxyfluorene	1.23±2.58	1.31±3.21	1.44±6.74	1.14±1.35	0.049
1-hydroxyphenanthrene	1.26±1.75	1.19±1.70	1.55±1.49	2.20±2.90	<0.001
2-hydroxyphenanthrene	0.37±0.40	0.43±0.44	0.52±0.53	0.71±0.68	<0.001
3-hydroxyphenanthrene	0.42±0.46	0.49±0.62	0.63±0.68	1.12±1.13	<0.001
4-hydroxyphenanthrene	0.65±1.09	0.61±0.91	0.78±4.01	0.57±0.67	0.864
9-hydroxyphenanthrene	1.12±1.70	1.21±1.42	1.29±1.58	1.22±1.08	0.002
1-hydroxypyrene	4.37±4.55	4.59±4.37	5.96±6.34	6.97±6.17	<0.001
HRV [median (25^th^, 75^th^)]†					
SDNN (msec)	39.85 (32.08, 50.63)	39.60 (31.70, 50.78)	39.30 (30.50, 47.85)	36.65 (28.10, 44.05)	0.013
rMSSD (msec)	23.60 (19.25, 30.90)	24.15 (19.03, 30.28)	22.70 (17.90, 28.60)	21.50 (17.25, 26.08)	0.002
LF (msec^2^)	362.54 (204.60, 637.49)	348.10 (194.96, 579.74)	334.04 (198.02, 640.99)	322.07 (179.00, 585.92)	0.068
HF (msec^2^)	142.53 (79.79, 291.94)	151.46 (83.07, 279.97)	129.41 (63.85, 228.75)	112.98 (51.28, 184.11)	0.001
TP (msec^2^)	1115.10 (701.36, 1787.95)	1096.45 (684.87, 1704.68)	1092.99 (597.18, 1769.85)	925.43 (516.89, 1665.19)	0.044

HF, high frequency; LF, low frequency; rMSSD, root mean square of successive differences in adjacent NN intervals; SDNN: standard deviation of NN intervals; TP: total power.

* Simple linear regression for the trend of urinary PAH metabolites with the exposure levels.

† Multivariate linear regression for the trend of HRV with the exposure levels with adjustment for age, sex, working years, smoking status, alcohol use, BMI, exercise and hypertension.

### Dose-dependent decrease in HRV associated with increased COEs

The mean levels of HRV indices in the control and low, intermediate, and high exposure groups are also shown in Table 3. HRV indices of workers in these three exposure groups were significantly lower than in the control group. Multivariate linear regression analyses revealed that elevated COEs levels were significantly associated with a decrease in SDNN, rMSSD, HF and TP (*P*_trend_ = 0.013, *P*_trend_ = 0.002, *P*_trend_ = 0.001 and *P*_trend_ = 0.044, respectively) after adjusting for age, sex, working years, smoking status, alcohol use, BMI, exercise and hypertension.

### The association between urinary PAH metabolites and HRV

**Figure 1:**
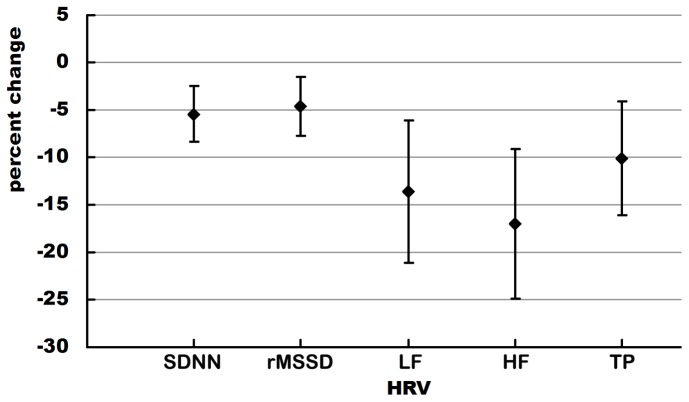
Percent change (95% CI) in HRV measures for an interquartile range increase in urinary 2-hydroxynaphthalene.

The geometric mean and quartiles of creatinine-adjusted concentrations of 10 urinary PAH metabolites among 1333 workers are shown in Table S2. The detection rate of the 10 PAH metabolites was >85%. 1-hydroxypyrene had the highest geometric mean, followed by 1-hydroxynaphthalene and 2-hydroxynaphthalene; 4-hydroxyphenanthrene had the lowest geometric mean. Because all workers had different levels of urinary PAH metabolites and there were significantly positive correlations between urinary PAH metabolites and the COEs levels, we stratified the workers by the quartiles of each urinary PAH metabolite level. Table 4 shows the associations between HRV and quartiles of each urinary PAH metabolite. After adjusting for age, sex, working years, smoking status, alcohol use, BMI, exercise and hypertension, decreased HRV indices (including SDNN, rMSSD, LF, HF and TP) were significantly associated with increased quartiles (from low to high) of urinary 2-hydroxynaphthalene (*P* = 0.005, *P* = 0.043, *P* = 0.006, *P* = 0.001 and *P* = 0.022, respectively and *P*_trend_<0.001, *P*_trend_ = 0.004, *P*_trend_ = 0.001, *P*_trend_<0.001, *P*_trend_ = 0.003, respectively), reduced LF was significantly associated with elevated quartiles of urinary 1-hydroxynaphthalene, and 9-hydroxyfluorene and 1-hydroxyphenanthrene (*P* = 0.047, *P* = 0.023 and *P*<0.001, *P*_trend_ = 0.004, *P*_trend_ = 0.017 and *P*_trend_<0.001, respectively). There was no other significant association between other urinary PAH metabolites and HRV indices (data not shown). Further analyses showed that there was a significant association between elevated quartiles of urinary 2-hydroxynaphthalene and decreased HRV, after adjusting for age, sex, working years, smoking status, alcohol use, BMI, exercise and hypertension (Figure 1). One interquartile range (1.81 µg/mmol creatinine) increase in urinary 2-hydroxynaphthalene caused 5.46% (95%CI, 2.50–8.32), 4.60% (95%CI, 1.44–7.66), 13.64% (95%CI, 6.14–20.39), 17.01% (95%CI, 9.15–24.05) and 10.13% (95%CI, 3.73–16.10) decrease in SDNN, rMSSD, LF, HF and TP, respectively.

**Table 4:** HRV indices of the workers stratified by the quartiles of each urinary PAH metabolite levels.

HRV [median (25^th^, 75^th^)]	OH-PAH Quartiles (µg/mmol creatinine)				*p*-Value*	*p*_trend_ †
	Q1	Q2	Q3	Q4		
1-hydroxynaphthalene	<0.94	0.94–1.57	1.58–2.89	>2.89		
SDNN (msec)	42.10 (33.05, 52.10)	39.40 (32.20, 50.60)	38.50 (30.70, 48.10)	36.90 (29.38, 46.95)	0.503	0.114
rMSSD (msec)	24.90 (19.65, 31.40)	23.60 (19.05, 30.05)	22.70 (18.40, 28.90)	22.50 (17.50, 29.15)	0.490	0.217
LF (msec^2^)	394.42 (216.76, 681.62)	350.75 (202.31, 624.99)	321.68 (197.95, 572.82)	320.90 (172.91, 596.73)	0.047	0.004
HF (msec^2^)	164.66 (91.56, 310.39)	146.61 (78.45, 249.59)	131.08 (69.24, 255.94)	115.84 (65.11, 235.74)	0.051	0.010
TP (msec^2^)	1193.80 (776.56, 1930.85)	1092.99 (671.68, 1843.74)	1024.84 (676.33, 1636.40)	1005.02 (570.28, 1661.76)	0.128	0.017
2-hydroxynaphthalene	<0.84	0.84–1.56	1.57–2.65	>2.65		
SDNN (msec)	42.50 (33.50, 52.40)	40.00 (32.05, 50.95)	38.80 (30.80, 48.50)	36.90 (29.38, 46.95)	0.005	<0.001
rMSSD (msec)	24.90 (19.90, 31.50)	23.60 (19.40, 30.85)	23.40 (18.10, 29.80)	22.50 (17.50, 29.15)	0.043	0.004
LF (msec^2^)	393.16 (221.30, 673.60)	365.96 (204.01, 624.99)	328.11 (193.06, 585.11)	320.90 (172.91, 596.73)	0.006	0.001
HF (msec^2^)	165.35 (97.92, 306.57)	157.54 (80.33, 297.99)	133.02 (69.66, 241.40)	115.84 (65.11, 235.74)	0.001	<0.001
TP (msec^2^)	1207.32 (762.25, 1926.99)	1121.93 (680.66, 1833.98)	1091.00 (656.79, 1697.38)	1005.02 (570.28, 1661.76)	0.022	0.003
9-hydroxyfluorene	<0.22	0.22–0.56	0.57–1.26	>1.26		
SDNN (msec)	39.50 (31.20, 49.45)	41.40 (33, 52.25)	38.40 (30.48, 48.05)	38.50 (30.85, 50.20)	0.105	0.215
rMSSD (msec)	23.20 (18.80, 30.15)	24.70 (19.70, 31.35)	22.90 (17.90, 29.13)	23.40 (18.40, 30.65)	0.145	0.459
LF (msec^2^)	376.12 (197.64, 707.11)	391.28 (223.18, 684.84)	323.84 (194.15, 550.36)	321.67 (183.07, 606.26)	0.044	0.017
HF (msec^2^)	155.74 (80.98, 294.58)	162.84 (87.27, 270.25)	126.40 (63.65, 234.23)	138.37 (78.71, 270.96)	0.016	0.082
TP (msec^2^)	1112.54 (671.68, 1841.37)	1198.97 (743.93, 1900.63)	1009.58 (610.14, 1657.31)	1079.73 (633.76, 1731.12)	0.124	0.100
1-hydroxyphenanthrene	<0.39	0.39–0.85	0.86–1.61	>1.61		
SDNN (msec)	40.30 (33.20, 51.00)	39.50 (31.60, 50.30)	39.90 (31.70, 50.50)	38.25 (30.23, 49.03)	0.436	0.192
rMSSD (msec)	25.00 (20.00, 31.50)	23.50 (18.10, 29.50)	23.50 (18.40, 31.00)	22.95 (18.10, 28.88)	0.023	0.067
LF (msec^2^)	367.03 (222.14, 676.25)	363.01 (197.95, 635.44)	346.56 (204.55, 609.43)	311.62 (168.25, 587.30)	0.265	0.050
HF (msec^2^)	163.77 (99.52, 335.74)	151.39 (76.18, 246.67)	132.54 (69.62, 264.54)	130.97 (63.61, 222.24)	<0.001	<0.001
TP (msec^2^)	1138.12 (762.25, 1889.49)	1109.55 (676.36, 1827.07)	1119.6 (650.41, 1782.46)	1011.98 (580.23, 1640.05)	0.280	0.047
1-hydroxypyrene	<1.85	1.85–3.37	3.38–6.10	>6.10		
SDNN (msec)	40.40 (32.00, 51.00)	38.50 (31.25, 47.45)	39.30 (30.70, 50.60)	39.70 (32.05, 51.30)	0.440	0.634
rMSSD (msec)	24.00 (19.70, 31.00)	23.35 (18.30, 29.43)	23.40 (18.10, 30.00)	23.70 (18.70, 31.10)	0.345	0.645
LF (msec^2^)	391.83 (225.20, 694.39)	325.79 (192.21, 549.88)	323.28 (184.79, 619.98)	358.78 (194.30, 659.46)	0.080	0.528
HF (msec^2^)	160.93 (88.67, 298.37)	138.68 (71.65, 237.76)	131.15 (66.63, 249.78)	146.14 (79.09, 297.39)	0.019	0.247
TP (msec^2^)	1131.41 (721.68, 1889.81)	1002.06 (684.83, 1602.69)	1105.20 (630.32, 1785.10)	1204.26 (691.20, 1797.23)	0.296	0.832

* Multivariate analysis of covariance for the differences between different groups with adjustment for age, sex, working years, smoking status, alcohol use, BMI, exercise and hypertension.

† Multivariate linear regression for the trend of HRV with the exposure levels with adjustment for age, sex, working years, smoking status, alcohol use, BMI, exercise and hypertension.

### Joint effects of working years and 2-hydroxynaphthalene levels on the decline in SDNN

**Figure 2:**
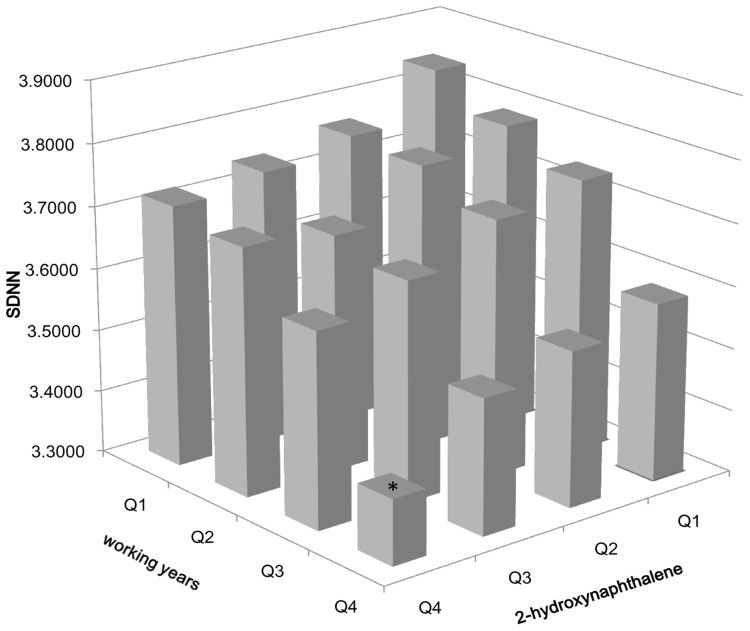
Joint effects of the quartiles of the working years and the quartiles of urinary 2-hydroxynaphthalene levels in SDNN. 2-hydroxynaphthalene: Q1 (<0.84 µg/mmol creatinine), Q2 (0.84–1.56 µg/mmol creatinine), Q3 (1.57–2.65 µg/mmol creatinine) and Q4 (>2.65 µg/mmol creatinine). The cutpoint (Q1–Q4) of the working years represent the quartiles of the working years in the quartiles of urinary 2-hydroxynaphthalene levels. Working years in Q1 of 2-hydroxynaphthalene levels: Q1 (<13.83years), Q2 (13.83–18.79years), Q3 (18.80–26.92years), and Q4 (>26.92years); working years in Q2 of 2-hydroxynaphthalene levels: Q1 (<15.42years), Q2 (15.42–19.42years), Q3 (19.43–27.75years), and Q4 (>27.75years); working years in Q3 of 2-hydroxynaphthalene levels: Q1 (<16.92years), Q2 (16.92–21.17years), Q3 (21.18–29.75years), and Q4 (>29.75years); working years in Q4 of 2-hydroxynaphthalene levels: Q1 (<15.92years), Q2 (15.92–21.17years), Q3 (21.18–29.83years), and Q4 (>29.83years). *P<0.01, compared with the group from Q1 of working years+Q1 of 2-hydroxynaphthalene levels. *P*_trend_ = 1.40×10^−4^.

SDNN reflects the overall variability of the heart rate and is an important determinant of cardiac autonomic function [5]. We further analyzed whether there was a cumulative effect of work duration and PAH metabolites on reduced SDNN. Figure 2 shows the combined effects of working years and 2-hydroxynaphthalene levels on the decrease of SDNN. As the number of years worked increased, SDNN gradually decreased in the same quartile of 2-hydroxynaphthalene levels (*P*_trend_ = 1.40×10^−4^). Compared with those in the first years-worked group and the first group of 2-hydroxynaphthalene levels, there was a significant decrease in SDNN among workers in the fourth group of working years and the highest group of 2-hydroxynaphthalene levels (*P* = 0.002). Moreover, it is evident that SDNN decreased the most among workers with the highest number of years worked in the highest quartile of 2-hydroxynaphthalene levels. However, the interaction term of working years and 2-hydroxynaphthalene levels was not significant in the relationship with SDNN (*P* = 0.284).

## Discussion

Few studies have examined the association between main coal-combustion-related pollutant exposure and altered HRV. Our results showed that occupational exposure to COEs was associated with a significantly dose-dependent decrease in HRV indices, including SDNN, rMSSD, HF and TP. In addition, there were inverse associations between urinary 2-hydroxynaphthalene levels and SDNN, rMSSD, LF, HF and TP of workers (*P*_trend_<0.01 for all), between 1-hydroxynaphthalene and 9-hydroxyfluorene and LF (*P*_trend_ = 0.004, *P*_trend_ = 0.017, respectively), and between 1-hydroxyphenanthrene and HF (*P*_trend_<0.001). SDNN decreased as the number of years of work increased in workers with similar internal exposure levels, which suggested that long-term exposure to PAHs further decreased HRV. More specifically, the largest reductions in SDNN were observed among the study populations with the longest number of years worked in the highest groups of urinary 2-hydroxynaphthalene levels. We concluded that the number of working years and 2-hydroxynaphthalene levels might jointly influence cardiac autonomic nerve function.

Our results provide strong evidence that occupational exposure to COEs, a type of coal-combustion-related air pollutant, results in cardiac autonomic dysfunction, implying that this type of air pollution may contribute to the increased global burden of cardiovascular disease. Ito et al. (2011) reported that coal-combustion-related components were associated with cardiovascular disease mortality and hospitalization in New York City [29]. The results from animal experiments showed that short-term exposure to coal-fired power plant emissions increased premature ventricular beat frequency in rats [30]. Our results suggested that reduced HRV may be an important mediator of the early adverse effects to the cardiovascular system caused by coal-combustion-related air pollution.

The carcinogenic potential of PAH exposure results in an increased risk of both lung cancer and cardiopulmonary mortality [22], [31]. However, it is far from clear what role PAHs play in contributing to early cardiovascular damage and disease. Based on the detection of all urinary PAH metabolites of each worker, we found that urinary 2-hydroxynaphthalene levels were significantly associated with reduced HRV, including SDNN, rMSSD, LF, HF and TP of workers. To our knowledge, there has been only one study exploring a negative association between PAHs exposure as measured by urinary 1-OHP and HRV in boilermakers [24]. The earlier investigation focused on the effects of oil-combustion-related pollutants on HRV and did not supply the other metabolic profiles of PAHs to assess pollution-specific effects. Therefore, we could not directly compare our results with their study of occupational exposure. However, findings from both epidemiological and toxicological studies on PAHs directly or indirectly support our results. A National Health and Nutrition Examination Survey study found that the highest tertile of urinary 2-hydroxynaphthalene, metabolite of naphthalene, was significantly associated with increased self-reported cardiovascular diseases (OR: 1.43) [32]. Some PAHs such as naphthalene have neurotoxicity, which may directly cause damage to the autonomic nervous system, leading to an imbalance in cardiac autonomic control [33]. An in vivo study showed that acute and prolonged naphthalene exposure can induce neuroendocrine disruption in rainbow trout [34]. Recent findings suggest that most of the PAHs and their metabolites can be found in the brain tissue of rats exposed to PAHs [35]. In addition, the direct effects of PAHs on cardiac ion channels or on activation of pulmonary neural reflex arcs may be another potential mechanism [1].

Our study has several strengths. First, our analysis was based on a large number of workers exposed to different levels of air pollutants; therefore, we were able to investigate a dose-effect relationship. Second, we not only monitored environmental airborne pollutants and 16 PAHs in different worksites, but also determined 10 urinary PAH metabolites for each worker, which provided a unique opportunity to calculate the association of each PAH metabolite with altered HRV. Finally, as most of the workers had worked at the same worksite for different periods of time, we estimated the joint or long-term effect of each urinary PAH metabolite and work duration on the HRV of workers with similar internal exposure levels.

Several limits had to be addressed. First, we did not use personal sampling to monitor individual environmental COE levels. However, urinary PAH metabolites of workers were significantly associated with exposure to environmental PAHs (Yang et al. 2007) [25]. Second, although the workers were mainly exposed to COEs during working hours, we could not exclude the effects on HRV of other sources of air pollution such as traffic air pollution, fossil fuel combustion and food PAHs. However, the results from the questionnaire data suggested that almost all workers lived in similar residential locations and thus the exposure levels to PAHs from other sources were also likely to be similar. Third, we only investigated the effects of individual PAH metabolites on HRV and did not assess the effects on HRV of other components of COEs such as SO_2_, CO and nitrogen dioxide (NO_2_). However, the concentrations of SO_2_, CO and NO_2_ were lower than the occupational exposure limit, except for the value of CO in the top coke oven. Fourth, monitoring of the 5-min HRV represents only short-term cardiac autonomic nerve modulation and HRV was measured only once because of the cross-sectional design. Nevertheless, the 5-min HRV was selected after excluding observations in the top 20% and bottom 20% of the 10 min HRV recording. Fifth, age, sex, BMI, smoking status and alcohol consumption may contribute to the metabolites of PAHs in coke oven workers. Aging causes decreased oxidation of xenobiotic materials [36]; body fat can affect the distribution of pyrene, and sex [36], smoking [37] and the use of alcohol [36], [38] may influence the P450 microsomial system. All these factors will change the hepatic metabolism of PAHs. However, we adjusted these potentially confounding factors in our models. Finally, we could not rule out residual and unmeasured confounders, although we adjusted for a wide range of confounders including age, sex, smoking, alcohol use, BMI, exercise and hypertension.

In conclusion, our study found that occupational exposure to COEs was significantly associated with cardiac autonomic dysfunction. In addition, PAH metabolites such as 2-hydroxynaphthalene are likely to play a role in disturbing cardiac autonomic function, implying that 2-hydroxynaphthalene may be an effective biomarker for assessing the effects of PAH exposure on cardiac autonomic function. We demonstrated that the dose-response decrease in HRV was most pronounced among coke oven workers with long years of employment, providing evidence of the cardiac toxicity of long-term exposure to COEs in the working environment. Our results further underline the importance of controlling occupational exposure to PAHs and surveillance of PAHs metabolites and cardiac autonomic function in workers, and will initiate further research on the possible mechanisms of HRV decline caused by PAHs. Although we do not have sufficient information to identify the normal values of HRV indices, our findings will have significant public health implications for the improvement of occupational safety among workers exposed to PAHs.

## Supporting Information

Figure S1 **Flow diagram.**
(TIF)

Table S1 Levels of environmental PAHs in different groups (mean ± SD).
(DOC)

Table S2 The distributions of urinary PAH metabolites of the workers (n = 1333).
(DOC)
