# Correction: The Dose-Response Decrease in Heart Rate Variability: Any Association with the Metabolites of Polycyclic Aromatic Hydrocarbons in Coke Oven Workers?

**DOI:** 10.1371/journal.pone.0133802

**Published:** 2015-07-20

**Authors:** Xiaohai Li, Yingying Feng, Huaxin Deng, Wangzhen Zhang, Dan Kuang, Qifei Deng, Xiayun Dai, Dafeng Lin, Suli Huang, Lili Xin, Yunfeng He, Kun Huang, Meian He, Huan Guo, Xiaomin Zhang, Tangchun Wu

There is an error in the first sentence of the Subject and methods subsection of the Material and Methods section. The correct sentence is: A total of 1,628 coke oven workers were recruited from a coke oven plant in Wuhan (Hubei, China) in 2010 and 1333 were included in this study.
