# Supplementary figures and images for: The Dose–Response Decrease in Heart Rate Variability: Any Association with the Metabolites of Polycyclic Aromatic Hydrocarbons in Coke Oven Workers?
Source: PLoS One. 2012 Sep 14;7(9):e44562. doi: 10.1371/journal.pone.0044562 (PMC3443084; doi:10.1371/journal.pone.0044562)

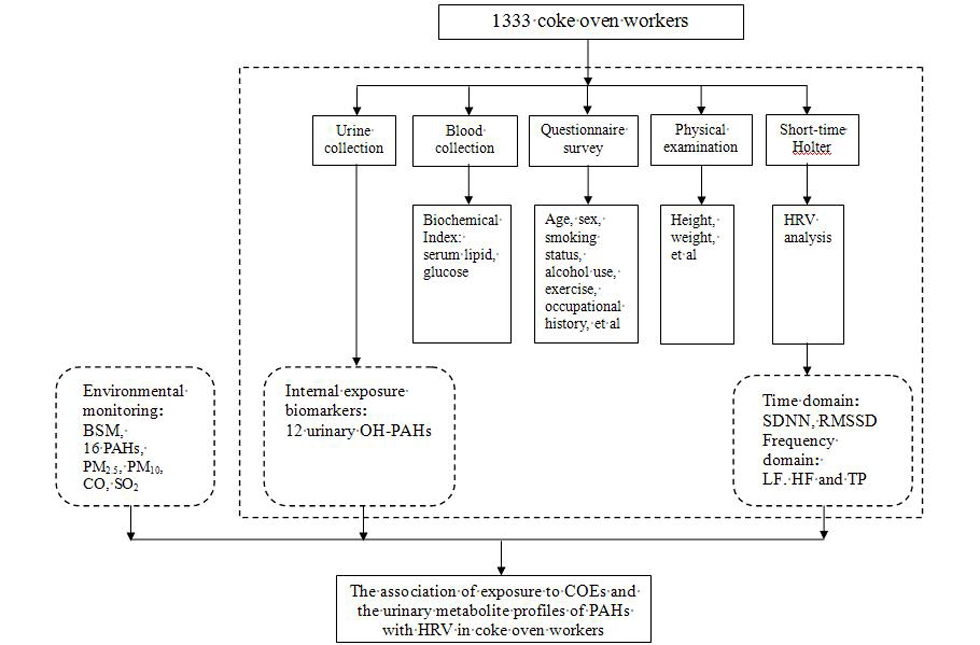

Supplement: Figure S1 — Flow diagram. (TIF) [file pone.0044562.s001.tif]
