# Supplementary material for: The Dose–Response Decrease in Heart Rate Variability: Any Association with the Metabolites of Polycyclic Aromatic Hydrocarbons in Coke Oven Workers?
Source: PLoS One. 2012 Sep 14;7(9):e44562. doi: 10.1371/journal.pone.0044562 (PMC3443084; doi:10.1371/journal.pone.0044562)
Supplement: Table S1 — Levels of environmental PAHs in different groups (mean ± SD). (DOC) [file pone.0044562.s002.doc]

| **Table S1.** Levels of environmental PAHs in different groups (mean ± SD). | | | | |
| --- | --- | --- | --- | --- |
| PAHs (μg/m^3^) | Office  (Control group, n = 5) | Coke oven (Exposure groups) | | |
|  |  | Adjunct workplaces  (Low, n = 6) | Bottom and side  (Intermediate, n = 16) | Top  (High, n = 15) |
| Naphthalene | 0.52 ± 0.06 | 0.71 ± 0.61 | 3.32 ± 2.11 | 31.24 ± 22.16 |
| Acenaphthene | 0.01 ± 0.00 | 0.31 ± 0.49 | 1.58 ± 2.25 | 13.45 ± 13.74 |
| Fluorene | 0.02 ± 0.00 | 0.07 ± 0.01 | 0.32 ± 0.23 | 2.36 ± 1.97 |
| Phenanthrene | < LOD | 0.04 ± 0.03 | 0.15 ± 0.10 | 1.47 ± 1.29 |
| Anthracene | 0.02 ± 0.01 | 0.04 ± 0.06 | 0.11 ± 0.07 | 0.78 ± 0.61 |
| Fluoranthene | 0.09 ± 0.11 | 0.63 ± 0.56 | 0.52 ± 0.40 | 5.16 ± 4.16 |
| Pyrene | 0.08 ± 0.03 | 0.11 ± 0.08 | 0.34 ± 0.26 | 3.69 ± 2.71 |
| Benz[a]anthracene^*^ | 0.01 ± 0.00 | 0.17 ± 0.14 | 0.31 ± 0.22 | 2.60 ± 2.05 |
| Chrysene^*^ | 0.04 ± 0.00 | 0.28 ± 0.23 | 0.53 ± 0.28 | 2.77 ± 2.09 |
| Benzo[e]pyrene^*^ | < LOD | 0.27 ± 0.07 | 1.16 ± 0.96 | 4.37 ± 4.17 |
| Benzo[b]fluoranthen^*^ | 0.03± 0.00 | 0.21 ± 0.16 | 0.55 ± 0.45 | 8.00 ± 14.38 |
| Benzo[k]fluoranthen^*^ | < LOD | 0.06 ± 0.01 | 0.28 ± 0.20 | 1.63 ± 1.35 |
| Benzo[a]pyrene^*^ | 0.05 ± 0.00 | 0.44 ± 0.07 | 0.58 ± 0.44 | 3.62 ± 3.16 |
| DB[ah]anthracene^*^ | 0.09 ± 0.12 | 0.22 ± 0.22 | 0.24 ± 0.27 | 2.05 ± 4.30 |
| B[ghi]perylene^*^ | 0.14 ± 0.17 | 0.05 ± 0.05 | 0.54 ±0 .48 | 3.12 ± 2.46 |
| I[123-cd]pyrene^*^ | 0.05 ± 0.00 | 0.11 ± 0.17 | 0.55 ± 0.69 | 3.99 ± 4.16 |
| PAHs: polycyclic aromatic hydrocarbons; LOD: limits of detection.  *known carcinogenic PAHs. | | | | |
