# Supplementary material for: The Dose–Response Decrease in Heart Rate Variability: Any Association with the Metabolites of Polycyclic Aromatic Hydrocarbons in Coke Oven Workers?
Source: PLoS One. 2012 Sep 14;7(9):e44562. doi: 10.1371/journal.pone.0044562 (PMC3443084; doi:10.1371/journal.pone.0044562)
Supplement: Table S2 — The distributions of urinary PAH metabolites of the workers (n = 1333). (DOC) [file pone.0044562.s003.doc]

| **Table S2.** The distributions of urinary PAH metabolites of the workers (n = 1333). | | | | | | |
| --- | --- | --- | --- | --- | --- | --- |
| PAH metabolites | Geometric  mean | Minimum | Percentile | | | Maximum |
| (μg/mmol creatinine) |  |  | 25th | 50th | 75th |  |
| Naphthalene metabolites |  |  |  |  |  |  |
| 1-hydroxynaphthalene | 1.66 | 0.04 | 0.94 | 1.57 | 2.89 | 65.29 |
| 2-hydroxynaphthalene | 1.49 | 0.05 | 0.84 | 1.56 | 2.65 | 49.72 |
| Fluorene metabolites |  |  |  |  |  |  |
| 2-hydroxyﬂuorene | 0.90 | 0.00 | 0.56 | 0.87 | 1.46 | 23.55 |
| 9-hydroxyﬂuorene | 0.38 | 0.00 | 0.22 | 0.56 | 1.26 | 107.71 |
| Phenanthrene metabolites |  |  |  |  |  |  |
| 1-hydroxyphenanthrene | 0.76 | 0.00 | 0.39 | 0.85 | 1.61 | 24.05 |
| 2-hydroxyphenanthrene | 0.29 | 0.00 | 0.19 | 0.30 | 0.54 | 4.06 |
| 3-hydroxyphenanthrene | 0.33 | 0.00 | 0.20 | 0.36 | 0.62 | 6.53 |
| 4-hydroxyphenanthrene | 0.22 | 0.00 | 0.13 | 0.33 | 0.71 | 64.24 |
| 9-hydroxyphenanthrene | 0.73 | 0.00 | 0.40 | 0.74 | 1.40 | 25.32 |
| Pyrene metabolite |  |  |  |  |  |  |
| 1-hydroxypyrene | 3.38 | 0.03 | 1.85 | 3.37 | 6.10 | 39.86 |
